# Effect of Photodynamic Therapy on Microorganisms Responsible for Dental Caries: A Systematic Review and Meta-Analysis

**DOI:** 10.3390/ijms20143585

**Published:** 2019-07-23

**Authors:** Analú Barros de Oliveira, Túlio Morandin Ferrisse, Raquel Souza Marques, Sarah Raquel de Annunzio, Fernanda Lourenção Brighenti, Carla Raquel Fontana

**Affiliations:** 1 School of Dentistry, Araraquara, São Paulo State University (UNESP), São Paulo 14801-903, Brazil; 2 School of Pharmaceutical Sciences, São Paulo State University (UNESP), Araraquara, São Paulo 14800-903, Brazil

**Keywords:** dental caries, photodynamic therapy, antimicrobial, microorganism, systematic and meta-analysis review

## Abstract

The aim of this study was to perform a systematic review of the literature followed by a meta-analysis about the efficacy of photodynamic therapy (PDT) on the microorganisms responsible for dental caries. The research question and the keywords were constructed according to the PICO strategy. The article search was done in Embase, Lilacs, Scielo, Medline, Scopus, Cochrane Library, Web of Science, Science Direct, and Pubmed databases. Randomized clinical trials and in vitro studies were selected in the review. The study was conducted according the PRISMA guideline for systematic review. A total of 34 articles were included in the qualitative analysis and four articles were divided into two subgroups to perform the meta-analysis. Few studies have achieved an effective microbial reduction in microorganisms associated with the pathogenesis of dental caries. The results highlight that there is no consensus about the study protocols for PDT against cariogenic microorganisms, although the results showed the PDT could be a good alternative for the treatment of dental caries.

## 1. Introduction

Dental caries is a hard dental tissue disease resulting from a chronic process that arises with the presence and interaction of factors such as microorganisms, diet, and host [1]. The most important factors for dental caries development is the interaction between a high sugar diet and specific oral bacteria within the oral biofilm. These bacteria produce acid through the fermentation of carbohydrates consumed by the host, which causes a sustained decrease in the oral cavity pH. Consequently, the enamel pH also reduces, causing its mineral dissolution [2]. If not properly treated, it may result in consequences for dental elements as well as chewing, talking, smiling, and on a patient’s life quality [3]. 

There are several available treatments for dental biofilm removal. These treatments include mechanical biofilm removal, antiseptics, and the use of chemoprophylactic agents [4]. However, the search for therapies that inhibit biofilm formation has led to significant research efforts to discover new treatments [5]. Photodynamic therapy (PDT) is as an effective tool in the treatment of various diseases [6] and a promising adjunctive treatment for dentin infection [7].

The PDT consists of a photosensitive molecule that absorbs an adequate wavelength light. This light-excited molecule, the photosensitizer (PS), can induce two reactions that may happen simultaneously (Type I and II reactions). In Type I reactions, the excited triplet PS reacts with biomolecules such as nucleic acids, lipids, and proteins by transferring an electric charge that produces radicals and radical ions. These radicals react with molecular oxygen to form reactive oxygen species (ROS) such as hydrogen peroxide, hydroxyl radicals, and superoxide anion. In Type II reactions, the PS in the excited triplet state transfers energy to the oxygen in the fundamental triplet state (process called the triplet–triplet annihilation), forming a singlet oxygen [8,9,10].

Scherer et al. (2017) [11] have recently proposed two new reactions, Types III and IV. In these reactions, the cytotoxic effect occurs even in the lack of oxygen in structures within the cells. Usually, Type III PSs are antioxidant carrier sensitizers (ACS) that are able to decrease the radical concentrations in the target cells and generate a singlet oxygen. In the Type IV reaction, the PSs cannot bind to the molecular target and after irradiation, a process called photoisomeration may occur. This process causes intramolecular remodeling that facilitates PS binding to the cellular target.

Considering that dental caries is a globally disseminated disease, the aim of this article was to conduct a systematic review of the literature in several databases, evaluate in vitro and in vivo studies, and the efficacy of PDT against microorganisms associated with dental caries etiology.

## 2. Results

### 2.1. Search Results

**Figure 1:**
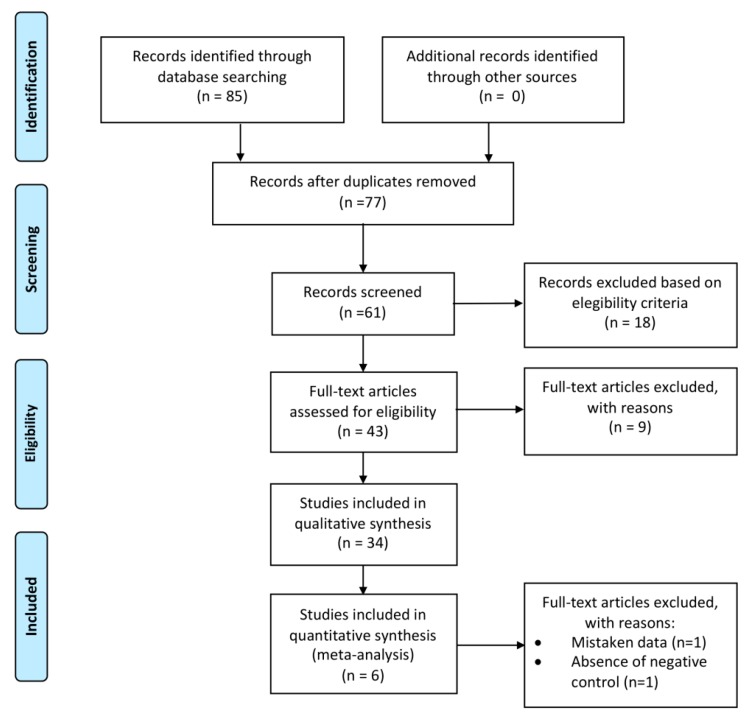
Flow diagram for our systematic review based on the PRISMA Guidelines.

The article selection process is summarized in the flow diagram presented in Figure 1. The initial electronic search yielded 85 articles. In total, eight duplicate articles were excluded; therefore, 77 papers remained in the study.

After title and abstract screening, 18 articles were excluded. Forty-three articles were eligible for full-text evaluation. Subsequently, 34 articles were included for qualitative analysis and four articles were included for quantitative synthesis (meta-analysis). From the 34 articles included for qualitative analyses, 30 studies were in vitro and four were randomized clinical trials. Table 1 summarizes the characteristics and results of the included articles.

### 2.2. Synthesis of Results

The systematic review showed that among the cariogenic microorganisms listed in the selected studies, the most studied microorganism was *Streptococcus mutans* (82%) [12,14,15,16,17,18,19,20,21,23,24,25,26,27,28,29,31,32,33,34,35,37,39,40,41,42,43,45]. 

The success of PDT depends on factors such as the administrated dose of light in the target cells and the time of exposure to light [46]. Considering these factors, we found that the most widely used light source was the red LED (32%) with wavelengths ranging from 625 to 670 nm [12,15,16,17,18,21,24,29,30,32,36,40], the most commonly used PS was methylene blue [13,16,18,21,23,24,27,28,29,30,32,33,36,38,39,42], and the most widely used pre-irradiation time was 5 min [12,14,15,16,17,19,20,21,22,27,28,30,31,32,33,39,42,43,45]. 

Regarding the ability to reduce the number of viable bacteria, most articles showed less than three logs of reduction [13,17,20,21,22,25,27,28,29,31,32,35,37,38,40,42,44,45]. The most commonly used control group was a negative control with no intervention. In addition, few studies have reported whether the biofilm inhibitory capacity of this treatment modality was tested [27,35,41,45].

### 2.3. Level of Evidence

According to the level of evidence (LoE) based on guidelines of the Oxford University Center for Evidence-Based Medicine [47], we noticed only four articles with level of evidence 1 and 28 articles with level of evidence 3. This difference between levels can be explained by different types of study and show the knowledge curve regarding photodynamic therapy on microorganisms associated with the pathogenesis of dental caries. Thus, there is a need to perform more randomized clinical studies in animals models and humans to increase the quality of scientific information.

### 2.4. Meta-Analysis

**Figure 2:**
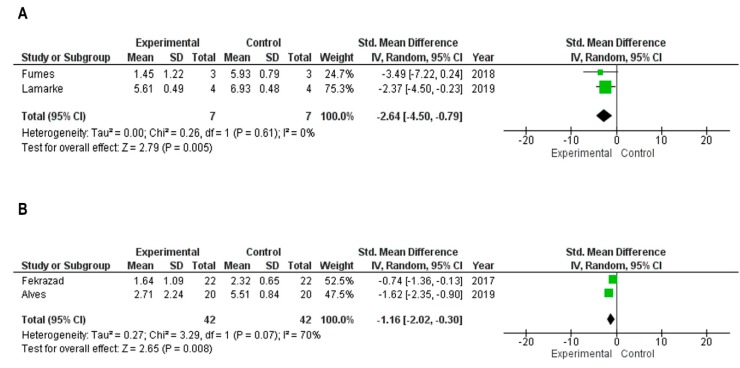
Results of the meta-analyses. The experimental group was formed based on colony forming units (CFU/mL) in the microorganisms that received photodynamic therapy (PDT). The control group was formed based on colony forming units (CFU/mL) in microorganisms that did not receive photodynamic therapy. (**A**) Meta-analysis in the in vitro study design. (**B**) Meta-analysis in the randomized clinical trials.

Only four studies were included in this analysis. Two of them among the in vitro studies [34,48] and more two studies related to randomized clinical studies [25,42]. One in vitro study was excluded due to a high standard deviation that was close to the mean of the data, which would possibly provide a non-parametric distribution of the data directly affecting the heterogeneity of the meta-analysis [45]. Furthermore, one other randomized clinical study was excluded due to the absence of the mean of the negative control [36]. Figure 2 illustrates the details about the statistical performance. Figure 2A shows the observed meta-analysis to the in vitro studies. The data showed a significant statistical difference to the experimental group that was formed by cariogenic microorganisms that received photodynamic therapy. The study of Lamarke et al. (2019) [44] presented more weight for analysis due to the larger sample size and lower standard deviation between the groups. Figure 2B shows the meta-analysis to the randomized clinical studies. Although there was a significant statistical difference to the experimental group, the heterogeneity among studies was *I*^2^ = 70%, which was considered too high to rely on the result of the statistical analysis. It is more likely that the heterogeneity found was due to the nature of the phenomenon evaluated for the type of study. 

## 3. Discussion

Dental caries is a multifactorial disease that slowly progresses in most individuals. In the absence of treatment, it can progress to oral pain and tooth loss [1]. 

Dental biofilm is one of the main local etiological factors of dental caries, and its mechanical removal through brushing with dentifrice associated with diet sugar reduction, is a method of control and prevention of the disease [49]. However, according to Valkenburg et al. (2016) [48], the efficacy of this method depends on the individual’s ability. Therefore, in some cases, as in special needs patients, this method needs complementary approaches. For this reason, the dental biofilm chemical control has been highly indicated. Chlorhexidine is known for its clinical and microbiological efficacy against various microorganisms present in the oral cavity [50]. However, its use has been questioned due to the adverse effects presented during its prolonged use [51]. 

Several studies have already demonstrated the susceptibility of cariogenic bacteria to photodynamic therapy [39,52,53], suggesting that this therapy may be useful as a minimally invasive adjuvant therapy for the control of dental caries [54] through cariogenic bacteria inactivation [55]. 

However, this therapy presents different challenges on the susceptibility of different microorganisms [56]. Most of the photosensitizers used in PDT are significantly more effective in inactivating Gram-positive bacteria than Gram-negative bacteria [57], which favors their use against dental caries microorganisms, since these caries lesions typically present the prevalence of Gram-positive strains [58]. 

For PDT to be successful, many variables should be considered such as the PSs used and the light dosimetry [59,60]. 

Among the evaluated articles, the most widely used photosensitizer (PS) was methylene blue (MB). This molecule belongs to the class of phenothiazine and presents solubility in water and ethanol. This PS efficiency in PDT is related to its intense absorption in the UV-visible region, whose maximum absorption wavelength is 664 nm, within the spectral region of 600 to 1000 nm (phototherapeutic window). It allows for the deep penetration of light in the biological tissues and expressive quantum yield for singlet oxygen formation [61,62]. The literature has already established the action of PDT mediated by MB, presenting its action against several bacteria associated with oral diseases [63,64]. MB has characteristics that promote good interaction with bacteria such as the positive charge on the molecule and low molecular mass. MB has action in both Gram-positive and Gram-negative bacteria, however, Gram-positive bacteria are more efficiently inactivated, due to the fact that the transport of positively charged molecules into the cell is facilitated. These bacteria have teichoic acids that give a negative charge to the outer surface [65], thus making this PS suitable for the inactivation of cariogenic microorganisms. 

Aside from MB, in the reviewed articles, the phenothiazine dye toluidine blue was the most widely used PS, followed by curcumin (a natural compound), rose Bengal, and green indocyanine, respectively. The data suggest that phenothiazine dyes have been the most investigated to date. Thus, these photosensitive agents might be promising for the adjuvant treatment of dental caries. However, more clinical studies with these PS should be developed to confirm this result.

Considering the pre-irradiation time [66], which is the period where the PS will remain in contact with the samples and may bind to the plasma membrane and/or internalize the target cells prior to light treatment, different times were evaluated. Andrade et al. (2013) [67] verified that in planktonic cultures of *Candida spp*. the photodynamic action was not dependent on the pre-irradiation time. However, for biofilms, a longer pre-irradiation time was required for the internalization of curcumin in the samples. In this review, the pre-irradiation time of the studies ranged from 1 to 30 min for different photosensitizers. Among the 34 articles, two did not report the time used, although this parameter is considered an essential information to determine clinical protocols in PDT. Fumes et al. (2018) [33] verified that 1-min pre-irradiation of the MB PS was able to reduce *S. mutans* in biofilm, and presented no statistical difference in the microbial load reduction when compared with superior times (2 and 5 min). In this same study, the authors reported the challenge of keeping a child with their mouth open for 5 min in a pilot clinical study, demonstrating the need to evaluate shorter times. Thus, studies evaluating shorter pre-irradiation times are desirable because they may develop clinical protocols that minimize patient discomfort.

Regarding the antimicrobial effect of PDT, there are several microbiological techniques that determine whether a substance can be considered bactericidal or potentially bactericidal. This determination can be influenced by factors such as microorganism growth conditions, bacterial density, test duration, and number of bacteria reduction. For a substance to be considered as a bactericide, it is necessary for a total inhibition of microorganism growth or ≤99.9% decrease in the initial inoculum (3-log 10 reduction in colony forming units [cfu]/mL) in the subculture [68]. From the 34 articles analyzed, only 11 presented a reduction greater than or equal to 99.9% [14,16,18,23,24,29,34,39,41,43,45]. This fact proves that eliminating these microorganisms is a great challenge, especially when they are in the biofilm.

The microorganisms present a great impact on public health, especially when in biofilm form, because they present a greater resistance to antibacterial agents and disinfection methods when compared to microorganisms in planktonic form [69]. Inhibition of biofilm formation may be relevant in cariogenicity reduction and in preventing the onset of new lesions [27,70].

Extracellular polysaccharides are the main constituents of cariogenic biofilms matrix, and are directly related with the virulence in biofilms [71]. Moreover, Zhao et al. (2013) [72] showed that these glue-like substances promoted the development of biofilm by conditioning the surface of the substrate. This indicates that the inhibition of the growth of the microorganisms is not the only strategy in reducing the development of dental caries. The influence on the expression of genes responsible for the polysaccharide synthesis and the reduction of this synthesis seem to be reasonable paths for further investigation [73]. 

Despite the notorious influence of polysaccharides on biofilm virulence, only three studies have evaluated it. Zanin et al. (2006) [12] analyzed the insoluble polysaccharide concentration in biofilms treated with the association of toluidine blue as a photosensitizer and a light-emission diode laser of 638.8 nm as the light source. The biofilms were evaluated at different times and it was concluded that in older biofilms, the concentration of insoluble polysaccharides was higher, indicating that despite the treatment, the age of the biofilm had an influence on the biofilm cariogenicity.

Gholibegloo et al. (2018) [35] evaluated the PDT influence on *gtfB* gene expression and concluded that there was a significant difference in the reduction of gene expression between the irradiated and non-irradiated groups, pointing to PDT as a potential treatment to prevent the formation of cariogenic biofilms. Nemezio et al. (2017) [28] concluded that PDT reduced the insoluble extracellular polysaccharide and intracellular polysaccharide concentration by nearly three- and four-fold, respectively, when compared to the control. Moreover, this effect resembled that of chlorhexidine. However, due to the lack of studies evaluating polysaccharides produced by biofilms, more studies are needed to prove the efficacy of photodynamic therapy in controlling the virulence of cariogenic biofilms.

Despite the time and number of studies involving PDT, few articles in this review had used in vivo models. In vitro studies have great importance for the initial analyses of treatments, however, when dealing with dental caries, it is important to emphasize that the oral cavity is composed of more than 700 microorganism species [74] and some of these species can be lost when in vitro biofilm models are used to mimic the oral environment. This limitation may be important to encourage new studies using in vivo models.

Among the vitro studies, the biofilm models were more frequent [12,13,15,18,19,20,21,24,27,28,31,32,33,34,35,37,38,39,40,43,44,45] than the suspensions, which confirm the fidelity of the data, since in biofilms, the microorganisms interact with each other and are more resistant to the antimicrobial agents when compared to the microorganisms in suspension [75]. Many studies have used monotypic biofilms of *S. mutans*, but this model is less representative of the oral environment and underestimates the complexity of the dental biofilm [76], so we emphasize the importance of studies with multispecies biofilms.

Limited clinical information remains on the use of PDT against cariogenic microorganisms. The appropriate parameters of energy dose, photosensitizer concentration, pre-irradiation time, and exposure should be developed through additional studies. 

## 4. Materials and Methods 

### 4.1. Eligibility Criteria

The systematic review was undertaken following the PRISMA (Preferred Reporting Items for Systematic Review and Meta-Analysis) guidelines [77]. The “PICO” strategy for systematic exploratory review guided the research question development [78]. This study aimed to answer the following question: Is photodynamic therapy effective against cariogenic microorganisms? The PICO strategy was: P (cariogenic microorganisms), I (photodynamic therapy), C (non-photodynamic therapy applied), and O (microbial reduction).

The inclusion criteria for our systematic review were: (i) All types of study design (in vitro, in situ, in vivo, randomized clinical trial, case cohort, and case control); (ii) Studies involving cariogenic biofilm models; (iii) Articles that evaluated the influence of photodynamic therapy on cariogenic microorganisms; and (iv) Articles published in English.

In this systematic review, the following study designs were not included: (i) Review articles, letters to the editor, personal opinions, book chapters, or conference abstracts; (ii) Studies that did not present a control group; (iii) Non-English language articles; and (iv) Articles where the full text was not freely available.

### 4.2. Search Strategy

Three independent examiners (ABO, RSM, and SRA) conducted an electronic search in the PubMed, Embase, SCOPUS, Lilacs, Science Direct, Web of Science, Medline, SCIELO, and Chochrane Library databases for articles published between December 1989 and March 2019. 

The following search terms and combinations were used: (((Photochemotherapy OR Photodynamic Therapy)) AND (Streptococcus mutans OR Caries OR Carious Dentin OR Caries disease)) AND Cariogenic Biofilm.

Based on the titles and abstracts of the studies, the three independent researchers selected the articles. The Mendeley Reference Manager Software^®^ was used to delete duplicate articles. 

### 4.3. Data Extraction and Analysis

The Preferred Reporting Items for Systematic Reviews and Meta-Analyses (PRISMA) statement was followed during the data assessment and extraction [77]. The following data were extracted from the studies: (a) type of study; (b) sample size; (c) time of pre-incubation of the photosensitizer; (d) photosensitizer; (e) ability to inhibit biofilm; (f) wavelength; (g) microorganism; (h) group control; and (i) reduction capacity. The Level of Evidence (LoE) for each study was determined according to the guidelines of the Oxford University Center for Evidence-Based Medicine [47]. 

### 4.4. Statistical Analysis

A meta-analysis was conducted using Review Manager 5.2 (Cochrane Collaboration). The effect size utilized was the standardized mean difference and the statistical analysis was performed using the random effect model. Two meta-analyses were realized due to the different types of study (randomized clinical studies and in vitro study). The *I*^2^ test evaluated the heterogeneity among the studies. A level of significance of 95% and level of reliability of 95% were chosen to perform the statistical analysis.

## 5. Conclusions

To date, photodynamic therapy has been suggested as a potential adjuvant to maximize the oral disinfection of microorganisms responsible for dental caries. However, additional studies are needed to determine the appropriate parameters for using this therapy as well as randomized and controlled clinical trials to verify the in vitro results in the in vivo models.

## Figures and Tables

**Table 1:** Summary of the characteristics of the included studies.

Study		Year	Study Design	Level of Evidence *	Sample Size	Irradiation Time **	Photosensitizer	Biofilm Inhibition	Wave-Length	Microorganism	Control Group	Biofilm Reduction (Log CFU/mL)
#1	Zanin et al. [12]	2006	In vitro	III	3	5 min	Toluidine blue	N/A	660 nm	*Streptococcus mutans*	Negative	<3
#2	Muller et al. [13]	2007	In vitro	III	9	1 min	Methylene blue	N/A	665 nm	Multispecies biofilm	Negative and chlorexidine digluconate 2%	<1
#3	Lutti Martin et al. [14]	2009	In vitro	III	N/A	1 min, 5 min, 15 min and 30 min	Fosfolipos and Hypericina	N/A	400 nm–505 nm	*Streptococcus mutans *and *Streptococcus subrinus*	Negative	3 (*S. subrinus*) and <3 (*S. mutans*)
#4	Mang et al. [15]	2012	In vitro	III	N/A	5 min	Porfimer sodium	N/A	630 nm	*Streptococcus mutans*	Negative	N/A
#5	Rolim et al. [16]	2012	In vitro	III	10	5 min	Methylene blue, Toluidine blue, Ortho and Malachite green	N/A	N/A	*Streptococcus mutans*	Negative	3
#6	Fekrazad et al. [17]	2013	In vitro	III	N/A	5 min	Toluidine blue, Radachlorine and Indocyanine green	N/A	660 mm and 810 nm	*Streptococcus mutans*	Negative	<3
#7	Spinei et al. [18]	2013	In vitro	III	N/A	N/A	Antocianine extract and methylene blue	N/A	625 nm–635 nm	*Streptococcus mutans, mitis, gordoni *and *sobrinus*	Negative	4.1
#8	Araujo et al. [19]	2014	In vitro	III	N/A	5 min	Curcumin	N/A	420 nm	*Streptococcus mutans *and *Lactobacillus acidophillus*	Negative	<1
#9	Manoil et al. [20]	2014	In vitro	III	12	5 min and 10 min	Curcumin	N/A	360 nm–550 nm	*Streptococcus mutans*	Negative	2
#10	Diniz et al. [21]	2015	In vitro	III	12	5 min	Methylene blue	N/A	660 nm	*Streptococcus mutans*	Negative	1.01
#11	Melo et al. [22]	2015	RCT	I	45	5 min	Toluidine blue	N/A	660 nm	Multispecies biofilm	Negative	<3
#12	Soria-Lozano et al. [23]	2015	In vitro	III	N/A	1 min/ 1 h/3 h	Methylene blue, Rose Bengal, and Curcumin	N/A	N/A	*Streptococcus mutans, Streptococcus sanguinis *and *Candida albicans*	Negative	6.0 (*Streptococcus *spp), 5.0 (*C.albicans*)
#13	Cintia Lima et al. [24]	2017	In vitro	III	N/A	10 min	Methylene blue	N/A	660 nm	*Streptococcus mutans*	Negative	>3
#14	Fekrazad et al. [25]	2017	RCT	I	22	1 min	Toluidine blue	N/A	630 nm	*Streptococcus mutans*	Negative	0.68
#15	Hyung-Jung et al. [26]	2017	In vitro	III	N/A	N/A	Curcumin and Curcuma xanthorrhiza extract	N/A	405 nm	*Streptococcus mutans*	Negative	>3
#16	Leili Beytollahi [27]	2017	In vitro	III	N/A	5 min	Methylene blue and Green Indocyanine	Yes	635 nm	*Streptococcus mutans*	Negative	<3
#17	Nemezio et al. [28]	2017	In vitro	III	4	5 min	Methylene blue	N/A	660 nm	*Streptococcus mutans*	NaCL solution 0.9% and chlorhexidine digluconate 0.12%	1
#18	Péres-Laguna et al. [29]	2017	In vitro	III	N/A	N/A	Methylene blue and Rose Bengal	N/A	N/A	*Streptococcus mutans*and *sanguinis*	Negative	6
#19	Azizi et al. [30]	2018	In vitro	III	6	5 min	Indocyanine green and Methylene blue	N/A	660 nm and 808 nm	*Lactobacillus acidophillus*	Chlorexidine digluconate 0.2%, NaOCL2.5% and Penicilin 6.3.3	N/A
#20	Darmani et al. [31]	2018	In vitro	III	N/A	5 min	Toluidine Blue	N/A	670 nm	*Streptococcus mutan, Streptococcus salivar, Lactobacillus casei *and* Actinomyces viscosus*	Negative	<1
#21	Esteban Florez et al. [32]	2018	In vitro	III	15	5 min	Methylene blue	N/A	660 nm	*Streptococcus mutans*	Negative and chlorexidine digluconate 2%	1,3
#22	Fumes et al. [33]	2018	In vitro	III	3	1 min, 2 min, and 5 min	Methylene blue	N/A	N/A	*Streptococcus mutans *and *Candida albicans*	Negative and chlorexidine digluconate 0.12%	<3
#23	Garcia et al. [34]	2018	In vitro	III	10	N/A	Fotoencitine and Photoditazine	N/A	660 nm	*Streptococcus mutans*	Negative and Methylene Blue	Complete eradication (Fotoencitine) and 6 (Photoditazine)
#24	Gholibegloo et al. [35]	2018	In vitro	III	3	5 min	Indocyanine green	Yes	N/A	*Streptococcus mutans*	Negative	<1
#25	Gomez et al. [36]	2018	RCT	I	10	3 min	Methylene blue	N/A	670 nm	*Aggregatibacter actinomycetemcomitans, Porphyromonas gingivalis, Prevotella intermedia *and* Tannerella forsythia*	US technique	N/A
#26	Míndez et al. [37]	2018	In vitro	III	9	2 min	Curcumin	N/A	455 nm	*Streptococcus mutans*	Negative	<3
#27	Oliveira et al. [38]	2018	In vitro	III	6	2 min	Methylene Blue	N/A	630 nm	Multispecies biofilm from saliva	Negative	<3
#28	Tokubo et al. [39]	2018	In vitro	III	3	5 min	Erythrosine and Methylene blue	N/A	N/A	*Streptococcus mutans*	Negative and chlorexidine digluconate 0.12%	4.3
#29	Trigo-Gutierrez et al. [40]	2018	In vitro	III	N/A	30 min	Cloroaluminium phthalocyanine nanoemulsion	N/A	N/A	*Candida albicans, Candida glabrata *and *Streptococcus mutans *	Negative	<3
#30	Alexandrino et al. [41]	2019	In vitro	III	N/A	N/A	Rose Bengal and Rose Bengal encapsulated with cyclodextrin	Yes	520 nm	*Streptococcus mutans*	NaCL solution 0.9% and chlorhexidine digluconate 0.12%	Complete eradication
#31	Alves et al. [42]	2019	RCT	I	20	5 min	Methylene blue	N/A	660 nm	*Streptococcus mutans*	Negative	2.8
#32	Esper et al. [43]	2019	In vitro	III	10	5 min	Hematoporfirine	N/A	420 nm and 480 nm	*Streptococcus mutans*	Negative	<1 (biofilm) and 3.8 and 6.78 (planktonic)
#33	Lamarke et al. [44]	2019	In vitro	III	4	2 min	Curcumin	N/A	420 nm	Multispecies biofilm	Negative and chlorexidine digluconate 0.12%	1.32
#34	Pourbajibagher et al. [45]	2019	In vitro	III	10	5 min	Cationic doped zinc oxide nanoparticle adhesive	Yes	435 nm	*Streptococcus mutans*	Negative	1.96

N/A: not available; min: minutes; h: hours; Negative: no treatment applied; * Level of evidence according to the Oxford Centre for Evidence-Based Medicine; ** Pre-irradiation time; RCT: randomized clinical trial.

## Abbreviations

PDT	Photodynamic Therapy
PS	Photosensitizer
ROS	Reactive Oxygen Species
ACS	Antioxidant Carrier Sensitizers
LoE	Level of Evidence
CFU	Colony Forming Units
MB	Methylene Blue
